# The impact of atosiban on pregnancy outcomes in women undergoing *in vitro* fertilization-embryo transfer: A meta-analysis

**DOI:** 10.1371/journal.pone.0175501

**Published:** 2017-04-19

**Authors:** Qian-Yi Huang, Min-Hua Rong, Ai-Hua Lan, Xiao-Miao Lin, Xing-Gu Lin, Rong-Quan He, Gang Chen, Mu-Jun Li

**Affiliations:** 1 Department of Reproductive Medical Research Center, First Affiliated Hospital of Guangxi Medical University, Nanning, China; 2 Research Department, Affiliated Cancer Hospital, Guangxi Medical University, Nanning, China; 3 Department of Children Rehabilitation Medicine, Guangxi Matemal and Child Health Hospital, Nanning, China; 4 Center of Genomic and Personalized Medicine, Guangxi Medical University, Nanning, China; 5 Department of Pathology, First Affiliated Hospital of Guangxi Medical University, Nanning, China; China Agricultural University, CHINA

## Abstract

**Background:**

Atosiban is administered to women undergoing in vitro fertilization-embryo transfer (IVF-ET) to improve pregnancy outcomes. However, the results of this treatment were controversial. We conducted this meta-analysis to investigate whether atosiban improves pregnancy outcomes in the women undergoing in vitro fertilization (IVF).

**Methods:**

Databases of PubMed, EMBASE, Web of Science, China BioMedicine, and Google Scholar were systematically searched. Meta-analyses were performed to investigate whether atosiban improves pregnancy outcomes in the women undergoing IVF.

**Results:**

Our results showed that atosiban was associated with higher implantation (OR = 1.63, 95% CI: 1.17–2.27; *P* = 0.004) and clinical pregnancy (OR = 1.84, 95% CI: 1.31–2.57; *P* < 0.001) rates. However, atosiban showed no significant association with the miscarriage, live birth, multiple pregnancy or ectopic pregnancy rates. When a further subgroup analysis was performed in the women undergoing repeated implantation failure (RIF), implantation (OR = 1.93, 95% CI: 1.45–2.57; *P* < 0.001), clinical pregnancy (OR = 2.48, 95% CI: 1.70–3.64; *P* <0.001) and the live birth (OR = 2.89, 95% CI: 1.78–4.67; *P* < 0.001) rates were significantly higher in the case group. Nevertheless, no significant difference was detected in the miscarriage and multiple pregnancy rates between the case and control groups.

**Conclusion:**

Atosiban may be more appropriate for women undergoing RIF and play only a limited role in improving pregnancy outcomes in the general population of women undergoing IVF. These conclusions should be verified in large and well-designed studies.

## Introduction

In vitro fertilization-embryo transfer (IVF-ET) is an assisted reproduction technology in which oocytes are extracted from an infertile woman and fertilized in vitro. The resulting embryo is then transferred into the uterine cavity [1]. This type of in vitro fertilization (IVF) research was conducted in animals as early as the 1930s by the Norfolk team, and the first human test-tube baby was born in 1978 [2]. Although tremendous progress has been made in the IVF-ET technique, the delivery and pregnancy rates associated with it remain low, at approximately 20% and 29%, respectively, in Europe and 32% and 39%, respectively, in the USA [3, 4]. Determining how the pregnancy rate can be increased and how improved pregnancy outcomes can be achieved are issues that need to be solved by every reproductive center. It is widely believed that the quality of the embryos and the conditions in the intrauterine environment play vital roles in the success of IVF-ET [5]. However, women who experience repeated implantation failure (RIF) do no achieve successfully pregnancies despite the use of high-quality embryos. Those failures may result in tremendous mental and economic pressure on these infertile couples. The definition of RIF is not standardized. It can be defined as a failure to achieve implantation in at least three consecutive IVF attempts when 1–2 high quality embryos were transferred in each cycle [6].

Atosiban is a receptor antagonist for vasopressin V1a and oxytocin. It selectively acts on the uterus to suppress uterine contractions and was initially used solely to prevent premature delivery [7]. In 2007, Pierzynski et al. were the first to use atosiban to facilitate IVF-ET in an animal model (rabbit). They also evaluated the effect of atosiban on human sperm motility in their study [8]. Their results indicated that clinical applications involving the application of atosiban for the proposed indications may be safe for embryos because it did not harm preimplantation rabbit embryo development or human sperm motility. The results of this embryo toxicity study encouraged Pierzynski et al. to proceed with a clinical experiment involving the use of atosiban in a woman with RIF in 2007 [9]. The woman was 42 years old and had a history of seven failed IVF-ET attempts. The atosiban decreased the patient’s uterine contractile activity and resulted in successful embryo implantation and a normal twin diamniotic pregnancy. Pierzynski et al. were therefore the first to apply atosiban in IVF-ET in a clinical setting. Since then, atosiban has gradually been increasingly applied in IVF-ET, and a similar result was achieved in a case report conducted by Liang et al in 2008 [10]. And the first prospective study which showed that atosiban may benefit subjects with RIF undergoing IVF/embryo transfer with cryopreserved embryos was conducted by Lan et al. in 2012 [11].

The impact of atosiban on pregnancy outcomes in women undergoing IVF has been investigated in recent years, and more efficient sets of parameters have been evaluated, including the implantation, clinical pregnancy, live birth, miscarriage, multiple pregnancy and ectopic pregnancy rates. However, the findings failed to reach a consensus [12–17]. Hence, we performed the current meta-analysis to provide a reference for clinical practices and to instruct future basic science and translational research. To the best of our knowledge, this is the first meta-analysis concerning the impact of atosiban on pregnancy outcomes in women undergoing IVF and RIF.

## Materials and methods

### Literature search

All relevant studies were identified using systematic searches of electronic databases (PubMed, EMBASE, Web of Science, China BioMedicine, and Google Scholar) by two atuthors (QYH and MHR). The last round of searching was conducted on January 20, 2017. The following search terms and strings were used: atosiban, in vitro fertilization-embryo transfer, IVF, repeated implantation failureand and RIF. Reference lists in identified articles and reviews were also searched manually to identify additional eligible studies.

### Inclusion and exclusion criteria

Publications that met all the following criteria were included: (1) the study compared the impact of atosiban and the placebo on pregnancy outcomes in the women undergoing IVF, and (2) the study was a clinical trial.

Publications that met all the following criteria were excluded: (1) the method used in the experimental design was not reasonable, (2) no usable data were reported, and (3) the report was a review or duplicate article.

### Data extraction

For each publication, three authors (AHL, XML, XGL) extracted the following information: the first author's name; the publication year; the country of origin; the participant type; the number of women in the case and control groups; the mean age of the women in each study; and the implantation rate, clinical pregnancy rate, live birth rate, miscarriage rate, multiple pregnancy rate and ectopic pregnancy rate. The data were independently extracted by two co-authors, and consensus was achieved via discussion among all authors if a disagreement occurred.

### Evaluation of study quality

The quality (validity) of individual trials was quantified by the Jadad scale [18] by two authors (QYH and MJL), using five criteria (one point each): (i) proper randomization, (ii) double blind, (iii) withdrawals documented, (iv) randomization adequately described, (v) blindness adequately described.

### Administration of atosiban in detail

Atosiban was used in the case group according to the instructions in all the 6 studies [12–17] included in the current meta-analysis. Atosiban is administered intravenously in three successive stage: an initial bolus dose (6.75 mg), performed with atosbian acetate injection 7.5 mg/ml (0.9 ml/vial), immediately followed by a continuous high dose infusion (loading infusion 300 ug/min) of atosbian acetate injection (5ml/vial, subsequent infusion 100 ug/min) up to 45 hours. The duration of the treatment should not exceed 48 hours. The total dose given during a full course of atosbian therapy should preferably not exceed 330 mg of the active substance. Intravenous therapy using the initial bolus injection should be started as soon as possible after diagnosis of preterm labor. Once the bolus has been injected, proceed with the infusion. In the case of persistence of uterine contractions during treatment with atosibian, alternative therapy should be considered.

In case of a re-treatment with atosibian is needed, it should also commence with a bolus injection of atosibian acetate injection 7.5 mg/ml (0.9 ml/vial) followed by infusion with atosibian acetate injection (5ml/vial).

### Statistical analysis

In all of the included publications, the term ‘implantation’ was congruously defined as the detection of at least a gestational sac in the uterus on transvaginal ultrasonic testing, and the implantation rate was defined as the number of gestational sacs divided by the number of transferred embryos. The term ‘clinical pregnancy’ was defined as the detection of a fetal heartbeat on transvaginal ultra-sound scanning approximately 2 weeks after a positive pregnancy test, and the clinical pregnancy rate was defined using the following formula: (the clinical pregnancy cases/the number of women in each group)×100%. The term ‘miscarriage’ was defined as the loss of a pregnancy before 20 weeks of gestation in all included publications, and the miscarriage rate was calculated using the following formula: (the number of miscarriage cases/the number of clinical pregnancy cases)×100%. The term ‘live birth’ was defined as a pregnancy that attained at least 25 weeks of gestational age, and the live birth rate was defined as (the number of live births/the number of women in each group)×100%. The term ‘multiple pregnancy’ was defined as two or more fetuses in one pregnancy at the same time, and the multiple pregnancy rate was defined as (the number of multiple pregnancy cases/clinical pregnancy cases) ×100%. The ectopic pregnancy rate was defined as (the number of ectopic pregnancy cases/the number of clinical pregnancy cases) ×100%. In our study, RIF was defined as the failure of implantation in at least two consecutive IVF attempts in which 1–2 high quality embryos were transferred per cycle.

To estimate differences in the pregnancy outcomes between case and control groups, we calculated the ORs and corresponding 95% CIs from the data. We also used Z-tests to evaluate the significance of the overall ORs. By convention, an observed OR > 1 was taken to imply a better pregnancy outcome in the atosiban group, and a corresponding 95%CI for the pooled OR that did not overlap 1 was considered to be statistically significant [19]. We judged heterogeneity by calculating the I^2^ statistic, with I^2^ ranges from 0–25% indicating low, 25–50% indicating moderate, and 50% indicating high heterogeneity [20]. When I^2^ < 50%, a fixed effects model was selected, whereas a random effects model was used when I^2^ > 50%. All statistical tests were performed using Review Manager 5.2 (Cochrane Collaboration, London, UK). Publication bias was assessed using Begg’s funnel plot, generated using Stata 12.0 (Stata Corp, College Station, TX, USA). *P* < 0.05 was considered statistically significant.

## Results

### Results of the literature search

**Fig 1:**
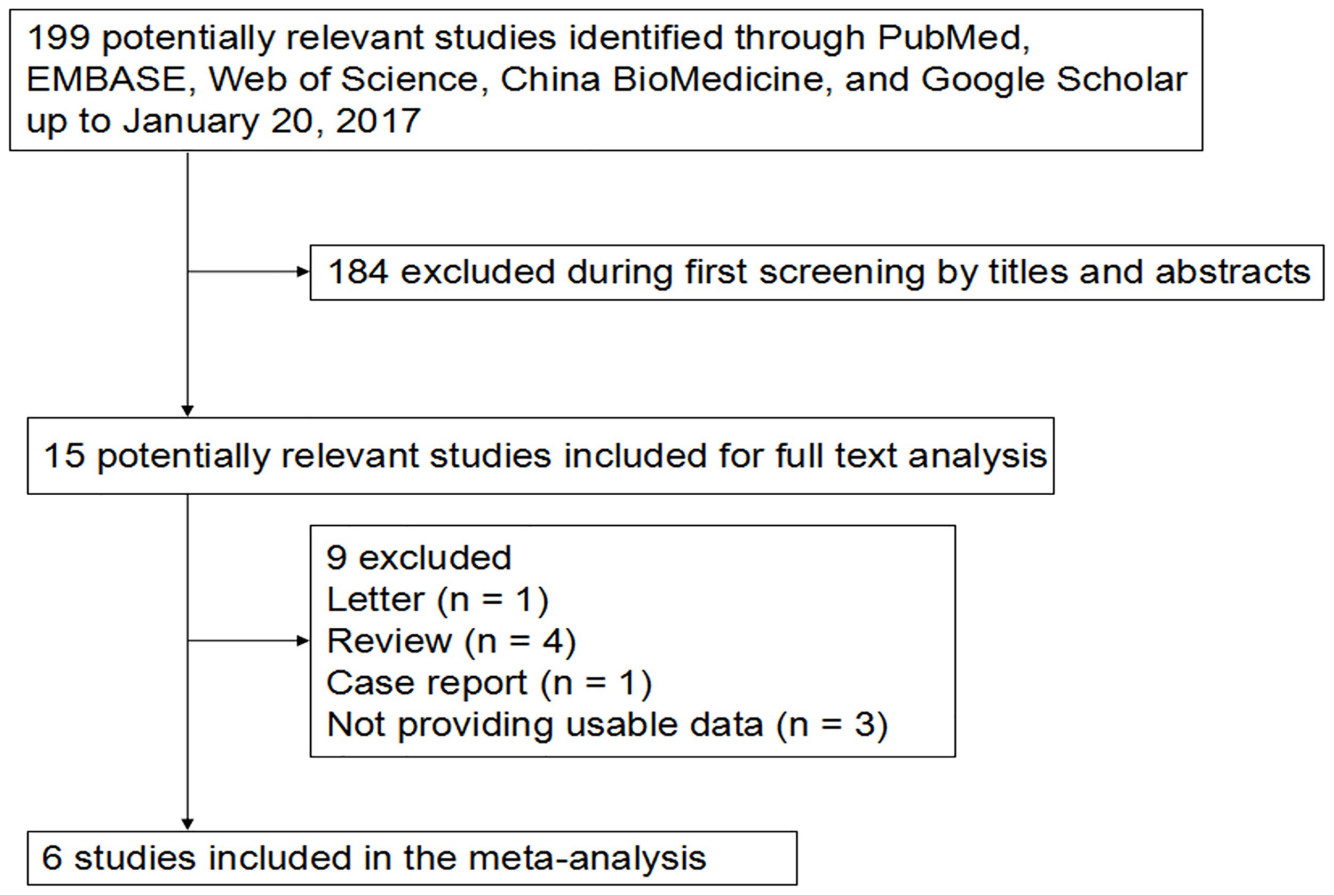
Flowchart.

Fig 1 is a flow diagram illustrating the search and selection criteria. A total of 183 potentially relevant studies were systematically identified in the databases of PubMed, EMBASE, Web of Science, China BioMedicine, and Google Scholar up to January 20, 2017. Of these, we excluded 168 studies based on review of titles and abstracts. An additional 3 studies were excluded because they were reviews (n = 4), letter (n = 1), case report (n = 1) or did not provide usable data (n = 3). In the end, the current meta-analysis involved six clinical trials [12–17] published from 2010 to 2014. Of them, three included studies [13–15] focused on RIF women who had experienced two or more consecutive IVF-ET attempts in which at least 1–2 high quality embryos were transferred per cycle. There was no restriction on the women in the remaining three trails [12, 16, 17], which included women who underwent all types of IVF procedures. The women who experienced RIF were chosen for further subgroup analysis. The minimum Jadad score of in those studies included was 1 and the maximum was 4. The characteristics of the studies included in this meta-analysis are summarized in Table 1.

**Table 1:** The characteristics and quality of the included studies.

Author (year)^Ref.^	Country	Research type	Participant type	Number of women		Overall IVF-ET outcomes	Case group	Control group	Jadad score*
				Case group	Control group				
Moraloglu et al. (2010) [12]	Turkey	RCT	All kinds of IVF	90	90	Implantation rate ^a^	20.4 (57/279)	12.6 (34/270)	2
						Clinical pregnancy rate ^a^	46.7 (42/90)	28.9 (26/90)	
						Miscarriage rate ^a^	16.7 (7/42)	24.4 (6/26)	
Chou et al. (2011) [14]	China	RT	RIF	70	80	Implantation rate ^a^	24.2(40/165)	11.8 (21/178)	1
						Clinical pregnancy rate ^a^	21.0 (21/70)	12.5 (10/80)	
						Miscarriage rate ^a^	9.5 (2/21)	20.0 (2/10)	
						Live birth rate ^a^	27.1(19/70)	10.0 (8/80)	
						Multiple pregnancy rate ^a^	33.3 (7/21)	10.0(1/10)	
Song et al. (2013) [13]	China	RCT	RIF	60	60	Implantation rate ^a^	30.0 (48/160)	20.3 (31/153)	1
						Clinical pregnancy rate ^a^	60.0 (36/60)	42.0 (25/60)	
						Miscarriage rate ^a^	5.6 (2/36)	16.0 (4/25)	
Zhang et al. (2014) [15]	China	RT	RIF	120	120	Implantation rate ^a^	27.0 (75/278)	16.4 (41/250)	1
						Clinical pregnancy rate ^a^	50.8 (61/120)	29.2 (35/120)	
						Miscarriage rate ^a^	8.2 (5/61)	11.4 (4/35)	
						Live birth rate ^a^	41.7 (50/120)	20.8 (25/120)	
						Multiple pregnancy rate ^a^	32.8 (20/61)	22.9 (8/35)	
						Ectopic pregnancy rate ^a^	3.3 (2/61)	8.6 (3/35)	
Ng et al. (2014) [17]	China	RCT	All kinds of IVF	400	400	Implantation rate ^a^	27.8 (278/999)	20.8 (264/1005)	4
						Clinical pregnancy rate ^a^	50.3 (201/400)	46.8 (187/400)	
						Miscarriage rate ^a^	18.4 (37/201)	18.7 (35/187)	
						Live birth rate ^a^	39.8 (159/400)	38.0 (152/400)	
						Multiple pregnancy rate ^a^	30.0 (65/201)	34.3 (68/187)	
						Ectopic pregnancy rate ^a^	4.5 (9/201)	5.3 (10/187)	
Chen et al. (2014) [16]	China	RT	All kinds of IVF	117	147	Clinical pregnancy rate ^a^	66.7 (78/117)	55.4 (82/147)	1
						Miscarriage rate ^a^	10.3 (8/78)	11.0 (9/82)	
						Multiple pregnancy rate ^a^	34.6 (27/78)	29.3 (24/82)	
						Ectopic pregnancy rate ^a^	2.6 (2/78)	0.0 (0/82)	

^a^ Values are expressed as percentages and the number of positive finding/the total number in the group

*Each study is rated according to its quality of bias-minimization using the Jadad scale, 0 (high bias) to 5 (low bias).

**Abbreviation**: Ref., Reference; RCT, randomized controlled trial; RT, retrospective study; IVF, in vitro fertilization; RIF, repeated Implantation failure; IVF-ET, in vitro fertilization-embryo transfer.

### Pregnancy outcomes of all the women undergoing in vitro fertilization

A total of 1754 women from six studies were included, including the women who experienced RIF and the women who underwent general IVF. Among these trials, in all of the case groups, atosiban was administered intravenously before and/or during embryo transfer. Placebo, saline, no medication or other interventions were applied in the control group.

**Fig 2:**
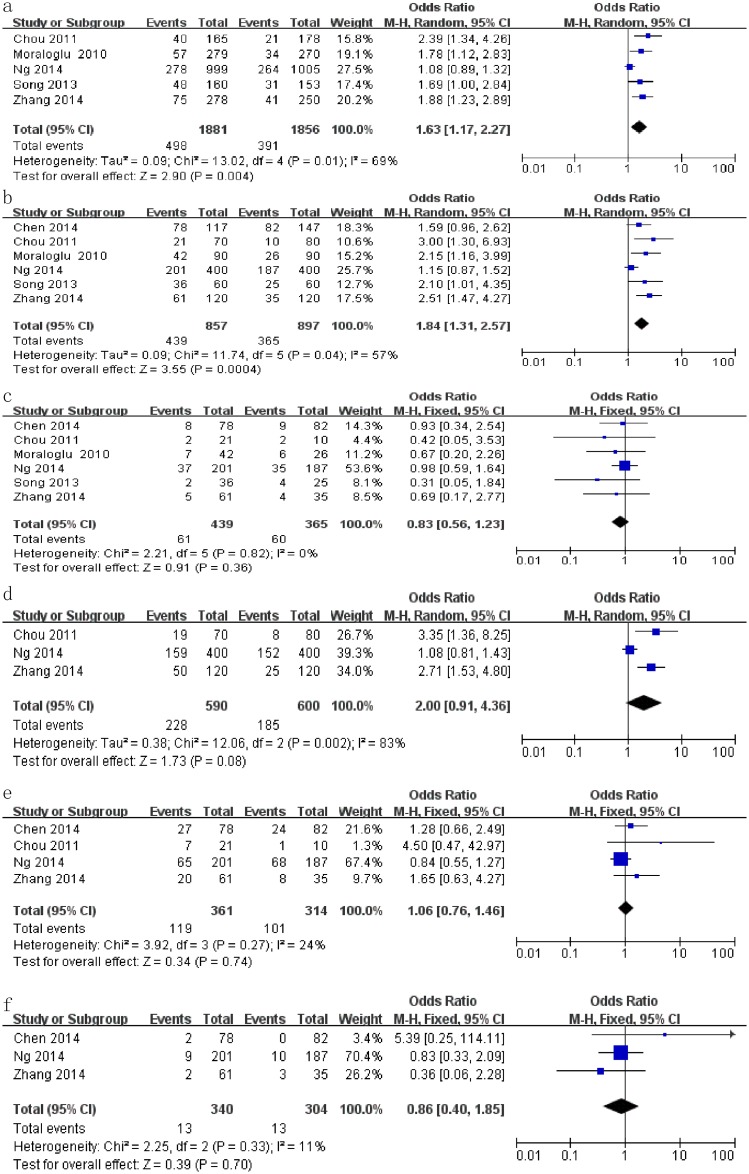
Forest plots for pregnancy outcomes in the women undergoing in vitro fertilization, including the implantation rate (a), clinical pregnancy rate (b), miscarriage rate (c), live birth rate (d), multiple pregnancy rate (e), clinical pregnancy rate (e), and ectopic pregnancy rate (f).

The impact of atosiban on implantation rate in the women undergoing IVF was assessed in five studies [12–15, 17]. Implantation rates were 26.5% (498/1881) in the case group and 21.1% (391/1856) in the control group (Table 2). Our results indicated that the use of atosiban was linked to a higher implantation rate in the women undergoing IVF, with an OR of 1.63 (95% CI: 1.17–2.27, I^2^ = 69%, *P* = 0.004, Fig 2a).

**Table 2:** Meta-analysis of all studies comparing pregnancy outcomes between case and control groups in the women undergoing IVF.

Pregnancy outcomes	Number of studies	Number of women	Positive/Total in case group ^a^	Positive/Total in control group ^a^	OR	(95%CI)	I^2^ (%)	*P*	Analysis model
Implantation rate	5	1490	26.5% (498/1881)	21.1% (391/1865)	1.63	(1.17–2.27)	69%	0.004	Random
Clinical pregnancy rate	6	1754	51.2% (439/857)	40.7% (365/897)	1.84	(1.31–2.57)	57%	<0.001	Random
Miscarriage rate	6	1754	13.9% (61/439)	16.4% (60/365)	0.83	(0.56–1.23)	0%	0.361	Fixed
Live birth rate	3	1190	38.6% (228/590)	30.8% (185/600)	2.00	(0.91–4.36)	83%	0.083	Random
Multiple pregnancy rate	4	1454	33.0% (119/361)	32.2% (101/314)	1.06	(0.76–1.46)	24%	0.737	Fixed
Ectopic pregnancy rate	3	1304	3.8% (13/340)	4.3% (13/304)	0.86	(0.40–1.85)	11%	0.700	Fixed

^a^ Values are expressed as percentages and the number of positive finding/the total number in the group.

**Abbreviation**: IVF, in vitro fertilization; OR, odd ratios; 95%CI, 95% confidence interval.

The impact of atosiban on clinical pregnancy rate in the women undergoing IVF was estimated in all of the selected studies [12–17]. The rates were 51.2% (439/857) in the case group and 40.7% (365/897) in the control group (Table 2). These results indicated that atosiban is associated with a higher clinical pregnancy rate in the women undergoing IVF, with an OR of 1.84 (95% CI: 1.31–2.57, I^2^ = 57%, *P* < 0.001, Fig 2b).

The impact of atosiban on miscarriage rate in the women undergoing IVF was estimated in all of the selected studies [12–17]. The rates were 13.9% (61/439) in the case group and 16.4% (60/365) in the control group (Table 2). These results showed that atosiban did not effectively decrease the miscarriage rate in the women undergoing IVF, with an OR of 0.83 (95% CI: 0.56–1.23, I^2^ = 0%, *P* = 0.361, Fig 2c).

The impact of atosiban on live birth rate in the women undergoing IVF was evaluated in three studies [14, 15, 17], in which 38.6% (228/590) in the case group and 30.8% (185/600) participants in the control group successfully had babies (Table 2). Our results suggested that atosiban was not associated with a higher live birth rate in the women undergoing IVF, with an OR of 2.00 (95% CI:0.91–4.36, I^2^ = 83%, *P* = 0.083, Fig 2d).

The impact of atosiban on the multiple pregnancy rate in the women undergoing IVF was evaluated in four studies [14–17]. The rates were 33.0% (119/361) in the case group and 32.2% (101/314) in the control group (Table 2). The results showed that there was no significant difference in the multiple pregnancy rate between the case and control groups in the women undergoing IVF and the OR was 1.06 (95% CI: 0.76–1.46, I^2^ = 24%, *P* = 0.737, Fig 2e).

The impact of atosiban on the ectopic pregnancy rate in the women undergoing IVF was reported in three studies [15–17]. The rates were 3.8% (13/340) in the case group and 4.3% (13/304) in the control group (Table 2). The results indicated that atosiban was not correlated with a lower ectopic pregnancy rate in the women undergoing IVF, with an OR of 0.86 (95% CI: 0.40–1.85, I^2^ = 11%, *P* = 0.700, Fig 2f).

### Pregnancy outcomes of all the women undergoing repeated implantation failure

Three of the included studies focused on women undergoing RIF (n = 510) who had experienced two or more consecutive IVF-ET attempts in which at least 1–2 high quality embryos were transferred in each cycle. Atosiban was applied in the case group, while the control group received no medicine.

**Fig 3:**
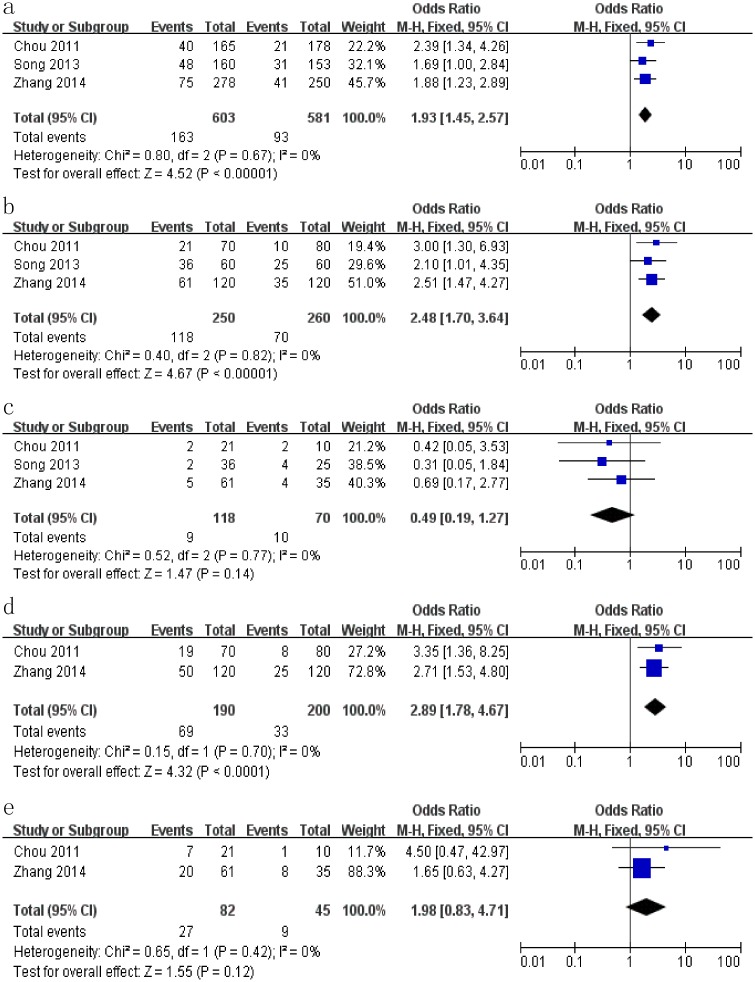
Forest plots for pregnancy outcomes in the women undergoing repeated implantation failure, including the implantation rate (a), clinical pregnancy rate (b), miscarriage rate (c), live birth rate (d), multiple pregnancy rate (e).

The impact of atosiban on implantation rate in the women undergoing RIF was assessed in three studies [13–15]. Implantation rates were (27.0%, 163/603) in the case group and (16.0%, 93/581) in the control group (Table 3). Our results indicated that the use of atosiban was linked to a higher implantation rate in the women undergoing RIF, with an OR of 1.93 (95% CI: 1.45–2.57, I^2^ = 0%, *P* < 0.001, Fig 3a).

**Table 3:** Meta-analysis of pregnancy outcomes between the case and control groups in the women undergoing RIF.

Pregnancy outcomes	Number of studies	Number of women	Positive/Total in case group ^a^	Positive/Total in control group ^a^	OR	(95%CI)	I^2^ (%)	*P*	Analysis mode
Implantation rate	3	510	27.0% (163/603)	16.0% (93/581)	1.93	(1.45–2.57)	0%	<0.001	Fixed
Clinical pregnancy rate	3	510	47.2% (118/250)	26.9% (70/260)	2.48	(1.70–3.64)	0%	<0.001	Fixed
Miscarriage rate	3	510	7.63% (9/118)	14.3% (10/70)	0.49	(0.19–1.27)	0%	0.141	Fixed
Live birth rate	2	390	36.3% (69/190)	16.5% (33/200)	2.89	(1.78–4.67)	0%	<0.001	Fixed
Multiple pregnancy rate	2	390	32.9% (27/82)	20.0%(9/45)	1.98	(0.83–4.71)	0%	0.122	Fixed

^a^ Values are expressed as percentages and the number of positive finding/the total number in the group.

**Abbreviation**: RIF, repeated Implantation failure; OR, odd ratios; 95%CI, 95% confidence interval.

The impact of atosiban on clinical pregnancy rate in the women undergoing RIF was estimated in three studies [13–15]. The rates were 47.2% (118/250) in the case group and 26.9% (70/260) in the control group (Table 3). These results indicated that atosiban is associated with a higher clinical pregnancy rate in the women undergoing RIF, with an OR of 2.48 (95% CI: 1.70–3.64, I^2^ = 0%, *P* < 0.001, Fig 3b).

The impact of atosiban on miscarriage rate in the women undergoing RIF was estimated in three studies [13–15]. The rates were 7.63% (9/118) in the case group and 14.3% (10/70) in the control group (Table 3). These results showed that atosiban did not effectively decrease the miscarriage rate in the women undergoing RIF in the women undergoing RIF, with an OR of 0.49 (95% CI: 0.19–1.27, I^2^ = 0%, *P* = 0.141, Fig 3c).

The impact of atosiban on live birth rate in the women undergoing RIF was evaluated in two studies [14, 15], in which 36.3% (69/190) in the case group and 16.5% (33/200) participants in the control group successfully had babies (Table 3). Our results suggested that atosiban was associated with a higher live birth rate in the women undergoing RIF, with an OR of 2.89 (95% CI:1.78–4.67, I^2^ = 0%, *P* < 0.001, Fig 3d).

The impact of atosiban on the multiple pregnancy rate in the women undergoing RIF was evaluated in two studies [14, 15]. The rates were 32.9% (27/82) in the case group and 20.0% (9/45) in the control group (Table 2). The results showed that there was no significant difference in the multiple pregnancy rate between the case and control groups in the women undergoing RIF and the OR was 1.98 (95% CI: 0.83–4.71, I^2^ = 0%, *P* = 0.122, Fig 3e).

### Sensitivity analysis

All the studies were from Chinese population except the one by Moraloglu et al. [12] which accounted for only a small portion of the sample. Thus sensitivity analysis was carried out by excluding the study by Moraloglu et al. [12] to re-evaluate whether atosiban improves pregnancy outcomes in the women undergoing IVF in Chinese population. However, the results were not altered (data not shown) in the subgroup meta-analysis of Chinese population, implying that our results based on all 6 studies were robust.

### Publication bias

**Fig 4:**
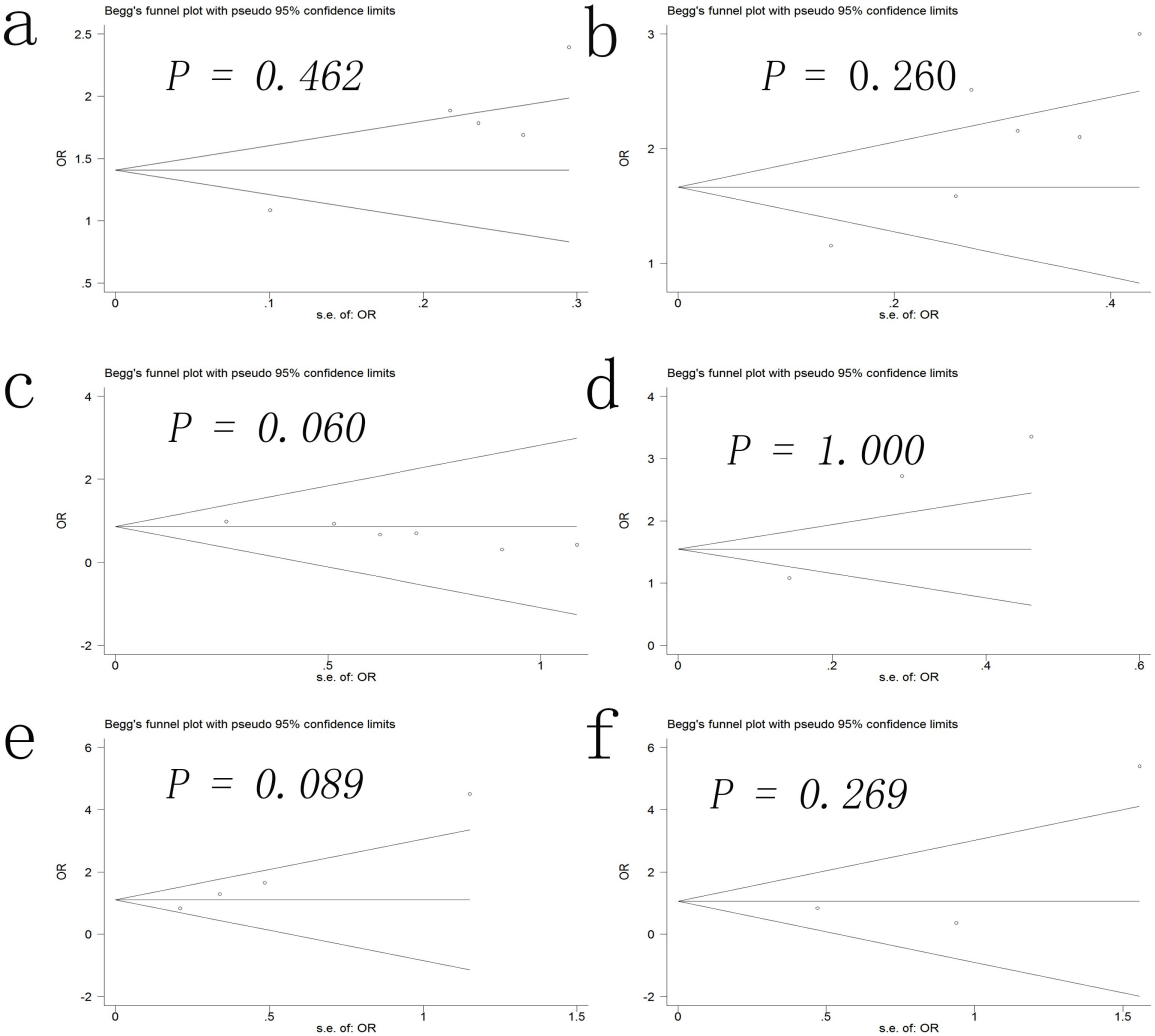
Begg’s funnel plot for publication bias of implantation rate (a), clinical pregnancy rate (b), miscarriage rate (c), live birth rate (d), multiple pregnancy rate (e), clinical pregnancy rate (e), and ectopic pregnancy rate (f) in the women undergoing in vitro fertilization.

**Fig 5:**
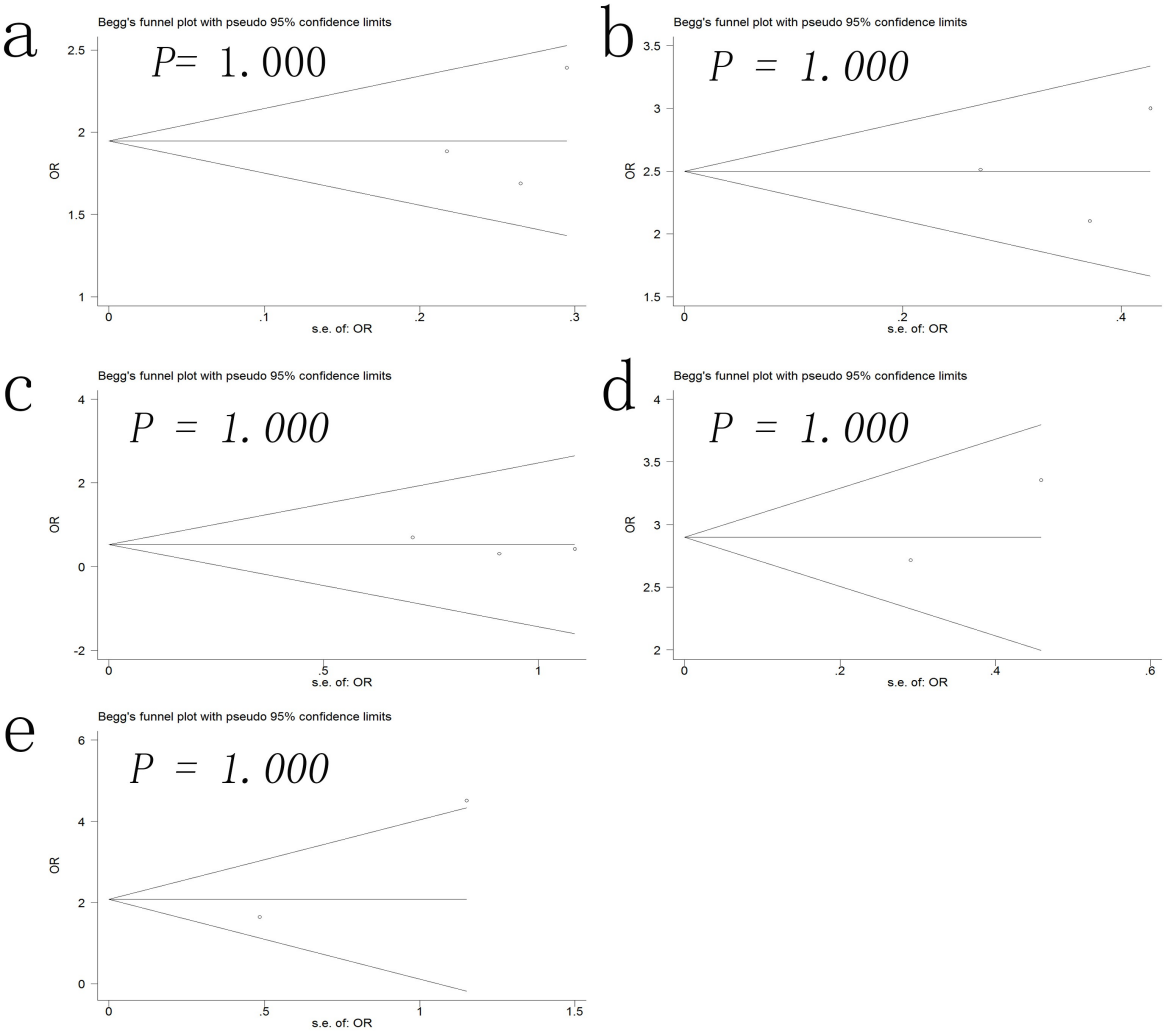
Begg’s funnel plot for publication bias of the implantation rate (a), clinical pregnancy rate (b), miscarriage rate (c), live birth rate (d), multiple pregnancy rate (e) in the women undergoing repeated implantation failure.

Begg’s funnel plot were performed to detect potential publication bias in this meta-analysis. No obvious asymmetry was observed in the pregnancy outcomes of all the women undergoing IVF (Fig 4a–4f) or RIF (Fig 5a–5e) in the Begg’s funnel plots.

## Discussion

Embryo transfer (ET) is the most crucial step in assisted reproductive technology, and the use of high-quality embryos and the presence of an optimal intrauterine environment are the basic determinants of embryo transfer success [21]. Fanchin et al. [22] demonstrated that uterine contractions occur during the course of embryo transfer. They verified that excessive uterine contractions can expel embryos from the uterus and that the frequency of uterine contractions was negatively correlated with implantation and clinical pregnancy rates. A study conducted by Spandofer et al. [23] showed that experiencing difficulty during the operation performed for embryo transfer had a negative influence on whether a successful pregnancy was achieved, whereas operations in which a smooth transfer was achieved had an increased pregnancy rate. According to Matorras et al. [24], the implantation rate and pregnancy rate will decrease as time required to transplant the embryo into the uterus increases and that an increase in uterine contractions is one of the mechanisms that affects this outcomes. Uterine contractions on the day of embryo transfer may dramatically decrease implantation and pregnancy rates. Therefore, physicians should aim to reduce the frequency of uterine contraction on the day of embryo transfer and the following days, and when standard manipulations are performed to transplant an embryo into the uterus, they should be performed as gently as possible. Additionally, oxytocin receptor antagonists should be applied to reduce the frequency and amplitude of uterine contractions.

Atosiban is a combined oxytocin/vasopressin V1A antagonist. It functions mainly by blocking oxytocin and vasopressin V1a receptors to decrease the frequency and amplitude of uterine contractions, which enhances implantation and pregnancy rates [25]. RIF can result from a broad array of embryonic, uterine, genetic, hematological and immunological causes. However, RIF remains unexplained in most cases, which results in considerable variation in how RIF is treated and managed. Strategies can include the intravenous administration of immunoglobulin and the application of atosiban [26]. In most women in our study, the cause of RIF was unknown. Atosiban did increase the live birth rate, but it was not effective in improving the live birth rate in the women undergoing IVF in our study. Similar results were reported by Ng et al. [17]. Atosiban interferes with prostaglandin F2a/oxytocin systems, which improves endometrial perfusion and may thereby increase the live birth rate [24]. Women suffering from RIF may be more prone to preexisting endometrial conditions than the general population of women who undergo IVF. Hence, applying atosiban may have a stronger impact in ameliorating endometrial conditions in RIF women. Atosiban was therefore found to be associated with a higher live birth rate in RIF women than in the general population of women undergoing IVF. More studies should be performed on this topic to validate these findings.

The objective of this meta-analysis was to evaluate the impact of atosiban on pregnancy-related outcomes in women undergoing IVF. We therefore collected data from six studies including 1754 women undergoing IVF, which consisted of 857 women who received atosiban and 897 women who received a placebo, saline or no medicine. We performed the meta-analysis by including all women undergoing IVF, and a further subgroup analysis was performed using women who suffered RIF in our study. Our results indicated that atosiban may be more appropriate for women undergoing RIF because it substantially enhanced the implantation, clinical pregnancy and live birth rates but had no significant impact on the miscarriage and multiple pregnancy rates in these women. However, atosiban may play only a limited role in improving pregnancy outcomes in the general population of women undergoing IVF because it was found to enhance the implantation and clinical pregnancy rates but not to enhance the live birth, miscarriage, multiple pregnancy and ectopic pregnancy rates in those women.

There are still some limitations to our meta-analysis. Firstly, the results may be affected by additional confounding factors, such as age, body mass index or duration of infertility et al., but some studies either did not report these baseline data or aggregated them in different ways, making it impossible to include them in the meta-analysis. Secondly, the intervention measures included placebo, saline and no medicine in the control groups of the studies included. Thus the differences between these factors may inevitably affect the results of the analyses in each study to some extent. Thirdly, three of the included articles were retrospective studies and the jadad score of four studies included was 1 [13–16], which may have data that are influenced by many unclear factors which may introduced instability to the results of our meta-analysis. Lastly, the sample of studies included in the meta-analysis was relatively small. Thus more randomized, double-blind comparison studies should be performed to confirm our findings.

In summary, the results of our study indicated atosiban may be more appropriate for women undergoing RIF because it substantially enhanced the implantation, clinical pregnancy and live birth rates but had no significant impact on the miscarriage and multiple pregnancy rates in these women. However, atosiban may play only a limited role in improving pregnancy outcomes in the general population of women undergoing IVF because it was found to enhance the implantation and clinical pregnancy rates but not to enhance the live birth, miscarriage, multiple pregnancy and ectopic pregnancy rates in those women. These conclusions should be verified in large and well-designed studies.

## Supporting information

S1 PRISMA NMA checklist (DOCX)
